# Acute Appendicitis Review: Background, Epidemiology, Diagnosis, and Treatment

**DOI:** 10.7759/cureus.8562

**Published:** 2020-06-11

**Authors:** Michael Krzyzak, Stephen M Mulrooney

**Affiliations:** 1 Internal Medicine, Staten Island University Hospital - Northwell Health, New York, USA; 2 Gastroenterology, Staten Island University Hospital - Northwell Health, New York, USA

**Keywords:** appendicitis, acute appendicitis, gastroenterology

## Abstract

Appendicitis is a common occurrence in both the adult and pediatric populations. The condition most commonly occurs between the ages of 10 and 20 years with a lifetime risk of 8.6% and 6.7% for males and females respectively. Its diagnosis focuses on clinical presentation and imaging modalities classified according to scoring systems such as the Alvarado scoring system. A number of imaging modalities can be used, with CT being the most common one. For acute appendicitis, surgical intervention is considered to be the gold standard of treatment. However, recent research has focused on other modalities of treatment including antibiotics and endoscopic retrograde appendicitis therapy (ERAT) to avoid surgical complications.

## Introduction and background

**Figure 1:**
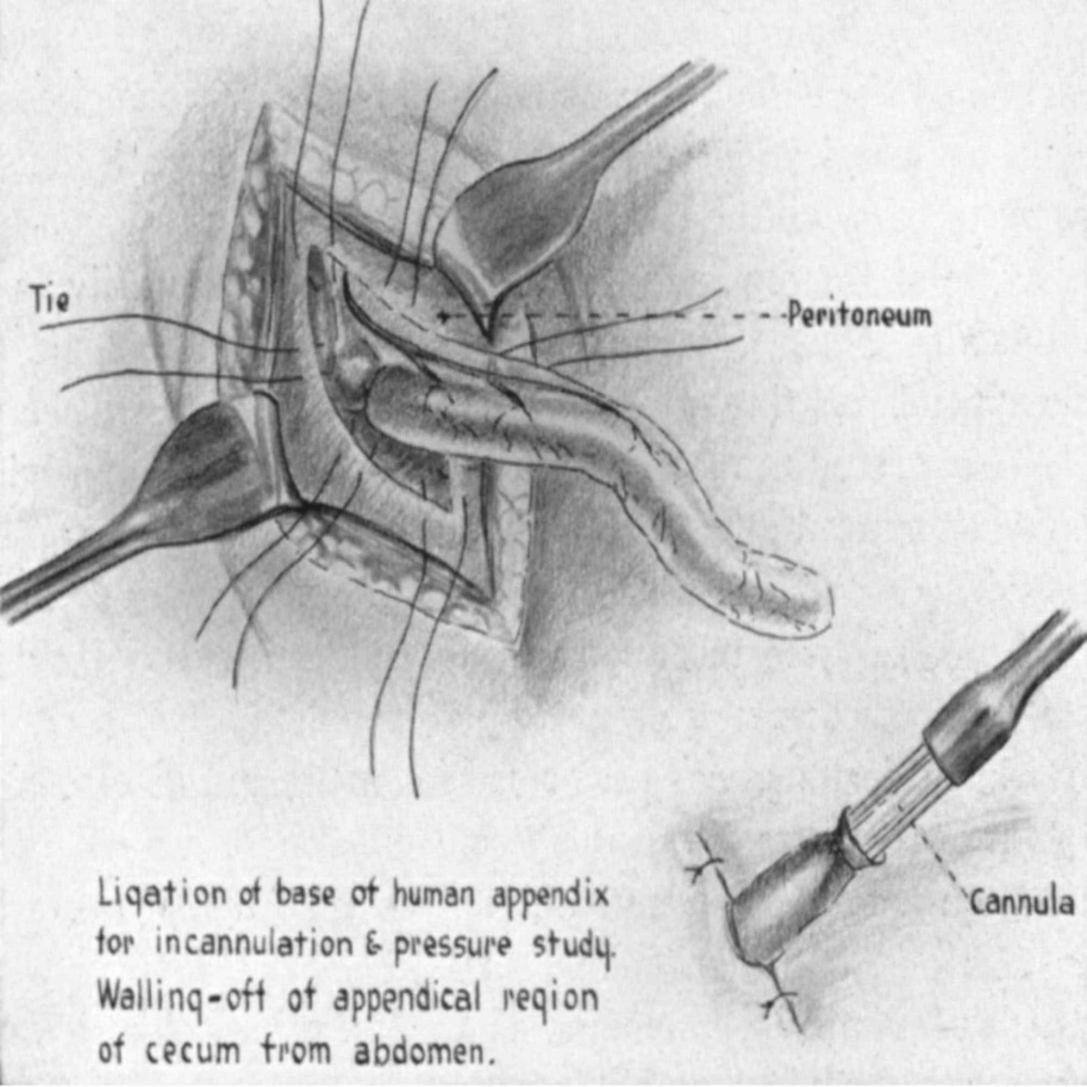
Manometric recording device* *[3]

The word appendicitis stems from Latin, combining appendix and -itis, and it means the inflammation of the appendix. The term appendix was coined in the 1540s to describe an elongated outgrowth of an internal organ [1]. Appendicitis was first described in 1759 by Metiever, but it was believed at the time that the appendix was not the origin of the disease process and it was termed perityphlitis, typhlitis, paratyphlitis, or extra-peritoneal abscess of the right iliac fossa [2]. From the early 20th century onwards, appendicitis originated from obstruction leading to the secretion of fluids by the appendix. An early study demonstrated, by inserting a manometric recording device (Figure 1), that higher pressures resulted in histologically evident hypercellularity and exudate pattern correlating with appendicitis [3]. Early mortality secondary to appendicitis was reported to be 26% [4].

The anatomy of the appendix has been described as narrow and long, passing upward behind the cecum, to the left behind the ileum and mesentery, or downward and inward into the pelvis. The average size is 1-9 inches. It is held by the mesentery and comprises three layers: organ sera, submucosa, and mucous [5].

From the early days onwards, the timeliness of diagnosis was considered to be critical to reducing mortality rates related to appendicitis. The clinical diagnosis was developed to determine if appendicitis is present. Charles McBurney labeled the precise spot to be 1.5-2 inches from the right anterior superior spinous process of the ilium on a line drawn to the umbilicus [4]. We now call this clinical sign the McBurney’s point.

## Review

Epidemiology

Appendicitis occurs most commonly between the ages of 10 and 20 years and it has a male-to-female ratio of 1.4:1. The lifetime risk is 8.6% for males and 6.7% for females in the United States [6]. Studies have indicated an association between acute appendicitis and the manifestation of colorectal cancer. In fact, 2.9% of patients who suffered from acute appendicitis were found to have colorectal cancer compared to 0.1% of those who did not [7]. In patients who are 55 years and older, acute appendicitis was found to be associated with right-sided neoplasm. The overall diagnosis of appendicitis, whether resected or treated conservatively, was associated with an overall increase in colorectal cancer rate. Hence, patients who are 55 years and older suffering from acute appendicitis should follow up to receive colorectal cancer screening [8].

Diagnosis

The initial presentation involves periumbilical colicky pain around the midgut. Localized pain coincides with the parietal peritoneum irritation. The pain intensifies over a period of 24 hours, accompanied by nausea, vomiting, and loss of appetite [6]. In 3.5% of appendicitis presentations, left iliac fossa deep palpation elicits pain in the right iliac fossa, which is termed Rovsing’s sign [9]. If the patient is found to have a positive Rovsing's sign, a barium swallow is then employed to confirm the diagnosis. Barium swallow was initially found to be 95% accurate [10].

Currently, diagnosis is made by helical CT and graded compression color Doppler ultrasonography [11]. A diagnosis can be made based on persistent right lower quadrant pain and a visualized appendix greater than 6 mm in diameter [12]. New studies point toward the efficacy of MRI, indicating 96-96.8% sensitivity and a 96-97.4% specificity [13,14]. Enabling this new modality will allow for patients such as children to avoid exposure to radiation and intravenous contrast medium, while still providing diagnostic accuracy. This finding foresees future first-line testing in children and possibly the general population.

The Alvarado scoring system is one of the most frequently used scoring systems to determine the need for surgical intervention for appendicitis (Table 1).

**Table 1:** Alvarado scoring system

Feature	Score
Migratory right iliac fossa pain	1
Nausea/vomiting	1
Anorexia	1
Tenderness in right iliac fossa	2
Rebound tenderness in right iliac fossa	1
Elevated temperature	1
Leukocytosis	2
Shift to the left of neutrophils	1

Scores of 1-4 indicate "discharged home", scores of 5-6 signify being "observed", and scores of 7-10 indicate the need to "undergo emergent surgery" [15,16]. The sensitivity and specificity of the Alvarado scoring system are reported to be 93.5% and 80.6%, respectively [17]. A simplified scoring system known as the Appendicitis Inflammatory Response scoring system involves eight variables (Table 2). These variables are vomiting, right-lower-quadrant pain, rebound tenderness, muscular defense, WBC count, proportion neutrophils, C-reactive protein (CRP), and body temperature [18].

**Table 2:** Appendicitis Inflammatory Response scoring system WBC: white blood cell; CRP: C-reactive protein

Feature		Score
Vomiting		1
Pain in right inferior fossa		1
Rebound tenderness or muscular defense	Light	1
	Medium	2
	Strong	3
Body temperature	>38.5 °C	1
Polymorphonuclear leukocytes	70–84%	1
	>85%	2
WBC count	10.0–14.9 x 10^9^/L	1
	≥15.0 x 10^9^/L	2
CRP concentration	10–49 g/L	1
	>50 g/L	2

Scores of 0-4 suggest "discharged home", scores of 5-8 mean being "observed", and scores of 9-12 indicate the need to "undergo surgery". In a study comparing the Appendicitis Inflammatory Response scoring system to the Alvarado scoring system, the sensitivity of the Appendicitis Inflammatory Response scoring system was found to be 93% compared to 90% with the Alvarado scoring system, with specificity reported to be 85% compared to 55%, respectively [19]. Other scoring systems have also emerged including Fenyo, Eskelinen, Tzakis, and Raja Isteri Pengiran Anak Saleha Appendicitis (RIPASA) [20].

Treatment

Early treatment of appendicitis focused on surgery. In 1883, Abraham Groves performed the first elective appendectomy [21]. In 1886, Reginald Fitz published the first paper describing early diagnosis and treatment of appendicitis [22]. In 1894, Charles McBurney described an incision parallel to the right rectus muscle oblique at approximately 1-4 inches [4]. This incision, known as the McBurney-McArthur muscle-splitting incision, was found to be associated with the lowest mortality [23]. Four advantages have been described with respect to using this technique: it provides easy direct access to the inflamed organ, drains can be placed laterally with sutures needed only on the peritoneum, the incision can be closed without risk of hernia, and, finally, access to cases of obstruction can be obtained without passing through additional structures [23].

During the mid-20th century, as surgical advances began to reduce complications, some studies examined whether surgery was necessary or whether a conservative route was safer and more efficacious [24]. Lower morbidity was found with a conservative route compared to the operative route [25]. Antibiotics were added to prevent infections. With bacillus coli being isolated from the appendix, the addition of a sulfonamide antibiotic was employed. Sulfanilamide was first used in 1940, and it was administered intraperitoneally as a local antibiotic. Mortality after five years was noted to be 0.4% [26]. Since 1959, studies have been examining the possibility of treatment with antibiotics solely. A 37% recurrence rate has been reported, indicating that antibiotics should be reserved for high-risk candidates [27].

In the 1990s, European investigators revisited the treatment of appendicitis by using antibiotics. It was found that 80% of preoperative diagnosis of appendicitis was correct with only one in six found to be having perforated appendicitis [28]. It is suggested that uncomplicated appendicitis may resolve with antibiotic treatment alone [29]. Reports show that appendicitis treated with antibiotics has a 91% success rate in the short term with 71% becoming appendectomy-free by one year [30]. In the United States, conservative management with antibiotics prior to surgical intervention has demonstrated positive results [31]. Forgoing or postponing surgical intervention enables treatment without surgical complications and have demonstrated patients being capable of an expedited return to work in comparison to surgical intervention [30,32].

Current guidelines continue to focus on early appendectomy. Uncomplicated appendicitis can be delayed in the hospital by 12-24 hours. On the other hand, early surgical intervention is thought to be associated with a lower risk of perforation [14]. Conservative treatment with antibiotics was found to be 18% less effective than surgical treatment [33]. Given substantial crossover in studies, it is recommended to continue to pursue surgical intervention as the first-line therapy [34]. Future studies employing different antibiotic regimens, both oral and intravenous, need to be conducted to examine the efficacy of antibiotics and explore the possibility of forgoing surgery for patients suffering from uncomplicated appendicitis [35]. Non-operative management has been found to have a high success rate of 86.1% [36]. On the other hand, the five-year recurrence of appendicitis in patients treated with antibiotics for acute appendicitis has been found to be 39.1% [37].

Other modalities are emerging as a treatment for acute appendicitis. Endoscopic retrograde appendicitis therapy (ERAT) employs endoscopic intervention in order to drain pus, extract fecalith, and stent when necessary. Of note, 93.8-95% of patients reported no recurrence following this method of treatment [38,39]. Laparoscopic appendectomy is another modality that enables same-day discharge; it was introduced by Semm in 1983 [40]. Patients who were discharged the same day after laparoscopic appendectomy were found to have lower rates of readmission compared with those who were hospitalized [41]. Other advantages include lower cost, lower risk of wound infections, and shorter recovery time [42-44].

## Conclusions

Appendicitis has been studied and treated for over a century. Diagnosis is based on imaging findings and clinical presentation. Currently, CT and graded compression color Doppler ultrasonography are generally employed to aid in the diagnosis. MRI has shown great promise as an alternative, with the added advantage of avoiding radiation exposure. Treatment is currently based on surgical intervention although future research looks to focus on more conservative measures such as antibiotics or other modalities. Antibiotic treatment has demonstrated efficacy in the short term but recurrence is likely in the long term. Some newer modalities of treatment have made it possible to forgo surgery by employing endoscopic intervention. Surgical advances with the use of laparoscopy enable same-day discharges, lower cost, fewer complications, and shorter recovery times.
